# Improving Patient Outcomes Through Sharing Best Practices

**DOI:** 10.1016/j.jaccas.2025.103789

**Published:** 2025-04-16

**Authors:** Sandra M. Oliver-McNeil

**Figure:**
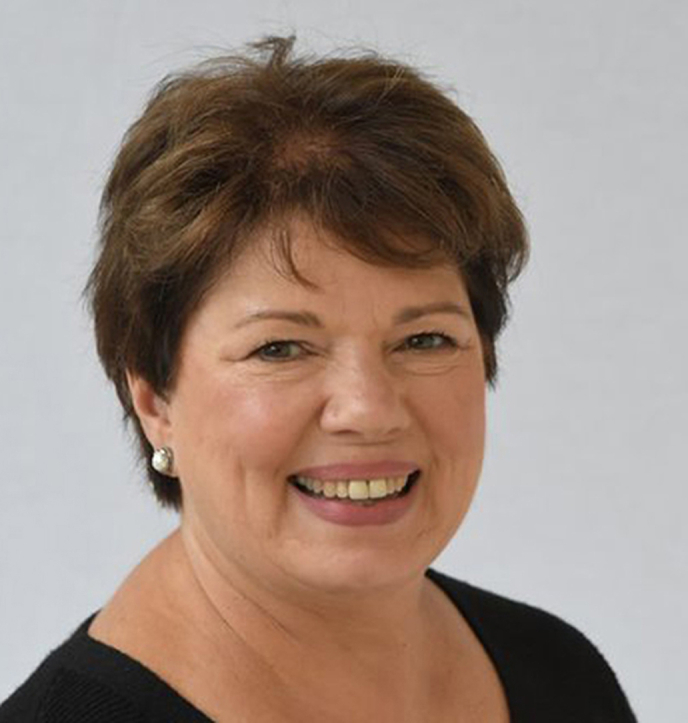

After graduating with my Bachelor of Science degree in nursing, I went to work in the medical/surgical intensive care unit of the local hospital a few miles from where I grew up. The design of the unit was more of a normal hospital floor, with 20 double rooms that had been converted to accommodate mechanical ventilation and a plan to add hardwired bedside monitors in 3 months. The timeline to get a staff, a third of whom were new graduate nurses, was fast and furious. The learning curve was steep, but the medical director was confident that this team of nurses and physicians would work together to be successful. Nurses were empowered to know everything about their patients, to know what potential complications patients might experience, and to communicate effectively with medical and surgical residents rotating through the unit. We learned from each other, and we shared knowledge with each other.

In the early 1990s I transferred to the cardiac catheterization laboratory as 1 of 3 registered nurses. It did not take me long to recognize that I was embarking on another new adventure. Angioplasty was defined as a controlled dissection, balloon pumps were the cornerstone of supporting circulation, and valvular heart disease was treated by valvuloplasty or surgical replacement with a mechanical ball-in-cage device. Bypass surgery was the treatment of choice for multivessel coronary disease, and the use of statins as secondary prevention was in its infancy. When coronary stenting started, stents had to be crimped onto the balloon by hand instead of at the factory, which sometimes resulted in retrieving the stent from another part of the body. High-dose heparin was used to prevent clotting but also resulted in bleeding complications from the femoral artery or vein. The concept of evidence-based practice was new because we were in the process of collecting data.

In the years that followed, the rapid development of technology and pharmaceuticals improved patient morbidity and mortality as well as quality of life. Stents were mounted onto balloons in the factory. Stent thrombosis became a clinical problem that resulted in the identification of genetic abnormalities where clopidogrel (Plavix) was not effective in a subgroup of patients. Impella and extracorporeal membrane oxygenation have provided other methods to provide circulatory support. Heath care institutions have developed specialty clinics to streamline patients with access to specialists in heart failure, structural heart disease, and arrythmias. Advanced practice providers, pharmacists, registered nurses, and allied health professionals have seen an increase in opportunities to treat patients within these specialty clinics to improve their adherence to medications and lifestyle modification. In addition, the team recognized that patients needed assistance to navigate the health care system and to cope with the challenges of health disparities.

Owing to the need to share best practices, *JACC: Case Reports* has developed a Cardiovascular Team Corner to highlight the challenges faced by advanced practice providers, pharmacists, registered nurses, allied health professionals, and trainees. This provides a platform to share how institutions have implemented quality improvement projects, developed specialty clinics, focused on lessons learned during the implementation process, and identified the issues they may face daily. Contributors to this corner may share what has worked in clinical practice, what are the pitfalls of setting up specialty programs, and how to overcome barriers. Successful implementation of programs requires the entire team to be supportive during the implementation period, but early recognition of which stakeholders need early buy-in can have an impact on success. Measurement of success can be direct or indirect financial gains, reduced hospital admissions or patient complications, staff retention, and patient satisfaction. *JACC: Case Reports* has provided the venue to share successful projects with other team members and institutions.

## Funding Support and Author Disclosures

The author has reported that she has no relationships relevant to the contents of this paper to disclose.

